# *In Situ* Analysis of a Silver Nanoparticle-Precipitating *Shewanella* Biofilm by Surface Enhanced Confocal Raman Microscopy

**DOI:** 10.1371/journal.pone.0145871

**Published:** 2015-12-28

**Authors:** Gal Schkolnik, Matthias Schmidt, Marco G. Mazza, Falk Harnisch, Niculina Musat

**Affiliations:** 1 Department of Dynamics of Complex Fluids, Max Planck Institute for Dynamics and Self-Organization, Göttingen, Germany; 2 Department of Environmental Microbiology, Helmholtz Centre for Environmental Research, Leipzig, Germany; 3 Department of Isotope Biogeochemistry, Helmholtz Centre for Environmental Research, Leipzig, Germany; University Hospital of the Albert-Ludwigs-University Freiburg, GERMANY

## Abstract

*Shewanella oneidensis* MR-1 is an electroactive bacterium, capable of reducing extracellular insoluble electron acceptors, making it important for both nutrient cycling in nature and microbial electrochemical technologies, such as microbial fuel cells and microbial electrosynthesis. When allowed to anaerobically colonize an Ag/AgCl solid interface, *S*. *oneidensis* has precipitated silver nanoparticles (AgNp), thus providing the means for a surface enhanced confocal Raman microscopy (SECRaM) investigation of its biofilm. The result is the *in-situ* chemical mapping of the biofilm as it developed over time, where the distribution of *cytochromes*, reduced and oxidized flavins, polysaccharides and phosphate in the undisturbed biofilm is monitored. Utilizing AgNp bio-produced by the bacteria colonizing the Ag/AgCl interface, we could perform SECRaM while avoiding the use of a patterned or roughened support or the introduction of noble metal salts and reducing agents. This new method will allow a spatially and temporally resolved chemical investigation not only of *Shewanella* biofilms at an insoluble electron acceptor, but also of other noble metal nanoparticle-precipitating bacteria in laboratory cultures or in complex microbial communities in their natural habitats.

## Introduction

*Shewanella* species are gram-negative facultative anaerobes, members of the class of electroactive bacteria, also known as exoelectrogens [1]. Electroactive bacteria can reduce extracellular insoluble electron acceptors (IEA), such as insoluble metal oxides and positively poised electrodes, as part of their respiratory chain [1–7]. They are therefore very important for metal cycling in nature, as they transform insoluble minerals, such as Fe_2_O_3_, into bioavailable ones, such as Fe(II) [4,5,7–12]. Recent fascination with electroactive bacteria has, however, stemmed from their emerging use as living catalysts in microbial electrochemical technologies (METs), such as microbial fuel cells (MFC) and microbial electrosynthesis (MES). In primary METs an electroactive biofilm is formed on an electrode, to be utilized for electricity production, wastewater purification, water desalination or the synthesis of chemicals such as alcohols, organic acids and fuels [1,13–16].

In oxygen-depleted environments *Shewanella oneidensis* MR-1, a frequently investigated *Shewanella* strain, can not only respire soluble electron acceptors, such as nitrate, Dimethyl sulfoxide (DMSO), fumarate and soluble metal ions [17], but also transfer its terminal respiratory electrons outside its outer membrane [6,9,11]. To do so, it employs three mechanisms: multi-heme *cytochromes*, nanowires and self-secreted electron transfer mediators (flavins) [18–23]. Of particular interest for this work are multi-heme *cytochromes*, which transfer respiratory electrons from the metabolic chain to adjacent bacteria, biofilm elements or the solid interface [23–27]. Lately it has emerged that these electron transfer cascades are flexible and that many of the involved components can play the role of a terminal electron donor [17,23,28]. Unlike other electroactive species, *Shewanella spp*. also produce and secrete flavins [23,27,29,30], which serve for electron cycling between the bacteria and the IEA. Reduced flavins are secreted, transported to the IEA and become oxidized. Oxidized flavins can then be transported back to the bacterium and accept more respiratory electrons [30,31]. Flavins are also cofactors of certain multi-heme *cytochromes* [23,32–34] and of other biomolecules [35,36]. They have also been reported to help solubilize IEA [37,38] and to serve as chemotaxis agents for *Shewanella* [39]. Therefore, flavins are expected to be particularly pertinent components for the *Shewanella* biofilm. As any other bacterial biofilm, *Shewanella* biofilms are also composed not only of cells, but to a great extent of extracellular polymeric substance (EPS), the main structural components of which are polysaccharides [40,41]. Alginate has been shown to be a common polysaccharide in EPS of wastewater bacterial communities [42], such as the gram-negative *Pseudomonas aeruginosa* [43]. It has been used before as a model EPS constituent for *Shewanella* cultivation [44]. Apart from polysaccharides, EPS in general and *Shewanella* EPS in particular have also been shown to contain *cytochromes*, flavins and nucleic acids [40,45].

One of the noteworthy properties of electroactive bacteria in general, and of *Shewanella spp*. in particular, is that they can precipitate silver nanoparticles (AgNp) when supplied with soluble silver salts in their growth medium [46,47], while still growing in spite of AgNp toxicity [47,48]. Noble metal nanoparticles, such as AgNp, can be used for Surface enhanced Raman spectroscopy (SERS), enhancing the Raman signal by up to six orders of magnitude in their immediate vicinity [49,50]. According to the SERS surface selection rules, only vibrational modes perpendicular to the particle surface become enhanced [49,50]. The enhancement is effective up to ca. 2 nm distance from the metal particle surface [49,50]. Silver or gold nanoparticles (AuNp) and roughened surfaces have become increasingly popular for use in SERS of biological samples, including both macromolecules [51–55] and whole cells [46,55–60]. Roughened Ag electrodes and AgNp/AuNp patterned microscope slides have been used as support for protein immobilization, cell deposition and biofilm growth [51–53,56,61,62]. Using a roughened or patterned support provides great surface selectivity, but results in the loss of accessibility to other areas in the vertical sample axis. An alternative approach for the investigation of bacteria and biofilms is to add soluble Ag(I) ions (e.g. in the form of AgNO_3_) to the growth medium, followed by a reducing agent, such as borohydride or citrate [57,59,60]. Thus AgNp are formed in the solution, in the biofilm, inside bacteria and on their outer membranes, providing Raman enhancement in different regions of the sample according to AgNp distribution [42,57]. It has been shown before that in some cases certain biomolecules, notably flavins, dominate the resulting signal [57,60,63], due to either adsorption of such molecules onto AgNp, or the location of AgNp precipitation, depending on the procedure employed. In order to clarify which biofilm components have contributed to the resulting spectra, some workers have acquired SERS spectra of the expected pure components under the same experimental conditions used for analyzing biological samples, and later compared the pure component spectrum to the biological sample spectrum [55,57], an approach that we shall also employ here.

In Confocal Raman microscopy (CRM), as in other types of confocal microscopy (see e.g. [64,65]), light reflected or scattered by the sample has to pass through a pinhole to be detected, thus providing spatially accurate detection, eliminating noise from positions outside the focal plane and improving lateral resolution beyond the light diffraction limit [66,67]. CRM combines confocal microscopy with Raman spectroscopy, resulting in an image where each pixel consists of a Raman spectrum. Summing over certain frequencies in the Raman spectra, one can preferentially detect specific vibrational transitions of interest in the image. Like SERS, CRM has also been gaining interest in recent years as a method of investigation for bacteria and bacterial biofilms [68–74]. By choosing an appropriate excitation wavelength, confocal resonance Raman microscopy can also be obtained [71–73].

In this work we present a new method combining SERS and CRM for the investigation of *S*. *oneidensis* MR-1 biofilms *in-situ* by surface enhanced confocal Raman microscopy (SECRaM), using bio-precipitated AgNp, formed by the bacteria as part of their anaerobic respiration process. We utilize this capability of *Shewanella* without resorting to the addition of Ag(I) salts, by simply allowing the bacteria to colonize a patch of biocompatible cured Ag/AgCl ink [75]. This way, we can follow the development of the undisturbed biofilm and its laterally resolved chemical composition over time under continuous anaerobiosis, while avoiding having to open the setup to add soluble Ag(I) salts or abrasive reducing agents. This approach stands in contrast also with Mass Spectrometry techniques, recently used for chemical analysis of biofilms and tissues [76–78], where the sample compartment must be opened or at least punctured, and where the sample is ablated for sampling. In this paper we report not only the temporal and spatial distribution of *cytochromes* in the biofilm, but also that of three other major biofilm components: flavins, polysaccharides and phosphate.

## Materials and Methods

### Cultivation

*Shewanella oneidensis* MR-1 (Zentrum für Angewandte Geowissenschaften, Universitaet Tuebingen) was used for all experiments. All growth media were prepared with autoclaved deionized water. All other aqueous solutions were prepared with Milli-Q water (Resistivity > 18 MΩ·cm). Pure cultures were stored at -80°C in glycerol stock. Liquid pre-cultures were prepared in 100 mL of Luria-Bertani broth (Roth, Karlsruhe, Germany), incubated aerobically 8 hours at 30°C with 150 rpm shaking and harvested during late exponential growth (OD_600_ = 1.5). Then 500 μL of the pre-culture was transferred into 100 mL of minimal medium [79] with 20 mM sodium lactate (Roth, Karlsruhe, Germany) as the substrate and no additional electron acceptor unless otherwise stated, and incubated aerobically for 15 h overnight at 30°C with 150 rpm shaking.

### Experimental setup

#### Microscope slide preparation for the different experiments

Standard microscope slides (Thermo Scientific, Braunschweig, Germany, for SECRaM) or coverslips (TH Geyer, Renningen, Germany, for SEM-EDX) were used as the sample support in all experiments, as follows: Ag/AgCl ink EXP 2642–15 (Creative Materials, Ayer, MA, USA) was used to paint a roughly elliptical patch (ca. 2x5 mm^2^) onto the substrate. The patch was then pre-cured at 100°C for 30 min and cured at 200°C for one hour. For the non-reducible ink control experiment (see below), a dielectric polymer ink 113–48 (Creative Materials, Ayer, MA, USA) was used for the patch instead of the Ag/AgCl ink, and was cured for one minute using UV light with post-curing at 160°C for one hour. All ink curing was performed under ambient atmosphere.

#### Bacterial deposition, setup sealing and its control

*S*. *oneidensis* MR-1 bacteria cultivated in minimal medium in mid-late exponential growth phase (OD_600_ = 0.6) were diluted to 50% with fresh minimal medium and deposited by pipette on the cured ink patch and its surroundings. A 25x25 mm^2^ coverslip was prepared by painting a 3 mm thick rim on one of its sides using a high precision synthetic brush dabbed in high-vacuum silicone grease (Dow Corning, Midland, MI, USA), as to create a spacer. Then the coverslip was fixed, with the greased side down, to the support glass, and pressed down to create a thin sealed chamber around the ink patch, containing a 25–40 μm thick layer of the bacterial suspension. Excess fluids were soaked with lint-free tissue paper (Kimberly, Koblenz, Germany) and a thick layer of high vacuum grease was used to cover the cants where the top coverslip met the support glass, to improve the seal and prevent the inflow of oxygen into the chamber. Six replicates were prepared: three on a microscope slide and three on a cover slip. To test the seal, the chamber was observed under an optical microscope at dark field configuration and 10× magnification. If a drift of bacteria and debris could be observed, this would be indicative of an air leak, and the sample would be discarded.

### Control experiments

(i) Abiotic control: to test whether Ag precipitate or light refracting structures would be formed in the chamber without the presence of bacteria, the chamber was sealed after depositing sterile minimal medium on the Ag/AgCl patch. (ii) Non-reducible ink control: to test whether the bacteria would create a biofilm on a patch not containing Ag/AgCl, a patch was prepared using dielectric polymer ink instead of Ag/AgCl ink, bacteria were deposited on it and the setup was sealed. (iii) Soluble electron acceptor control: to test whether the bacteria would create a biofilm on a Ag/AgCl patch in the presence of an alternative electron acceptor, 20 mM disodium fumarate was added to the bacterial culture before depositing the bacteria on the patch. Each control experiment was performed in duplicates.

### Visible microscopy and photography

An Olympus BH-2 optical microscope (Olympus, Hamburg, Germany) with a 10× air objective and an Olympus XC30 RGB video camera were used to optically follow biofilm growth in all samples. Images and videos were acquired at the dark field mode, where the bacteria appear as bright dots over a dark background.

### Confocal Raman Microscopy

Surface Enhanced Confocal Raman Microscopy (SECRaM) was performed on a WITec alpha300 confocal Raman microscope, using a 50 μm pinhole and a Zeiss LD plan-NEOFLUAR 20×/0.4 corr air objective with coverslip correction. 20× magnification was used in order to keep as many bacteria as possible in the focal plane while still resolving individual bacteria. The excitation wavelength was 532 nm, with laser power of 3 mW at the focal plane. The Raman detector was a newton EMCCD camera cooled to -60°C with a 600 g/mm grating. Integration time was 1 second and lateral resolution 2 pixel/μm. For a typical biofilm sample a time series was performed, and it was analyzed 1, 3, 6, 9 and 35 days after the samples were sealed.

**Fig 1:**
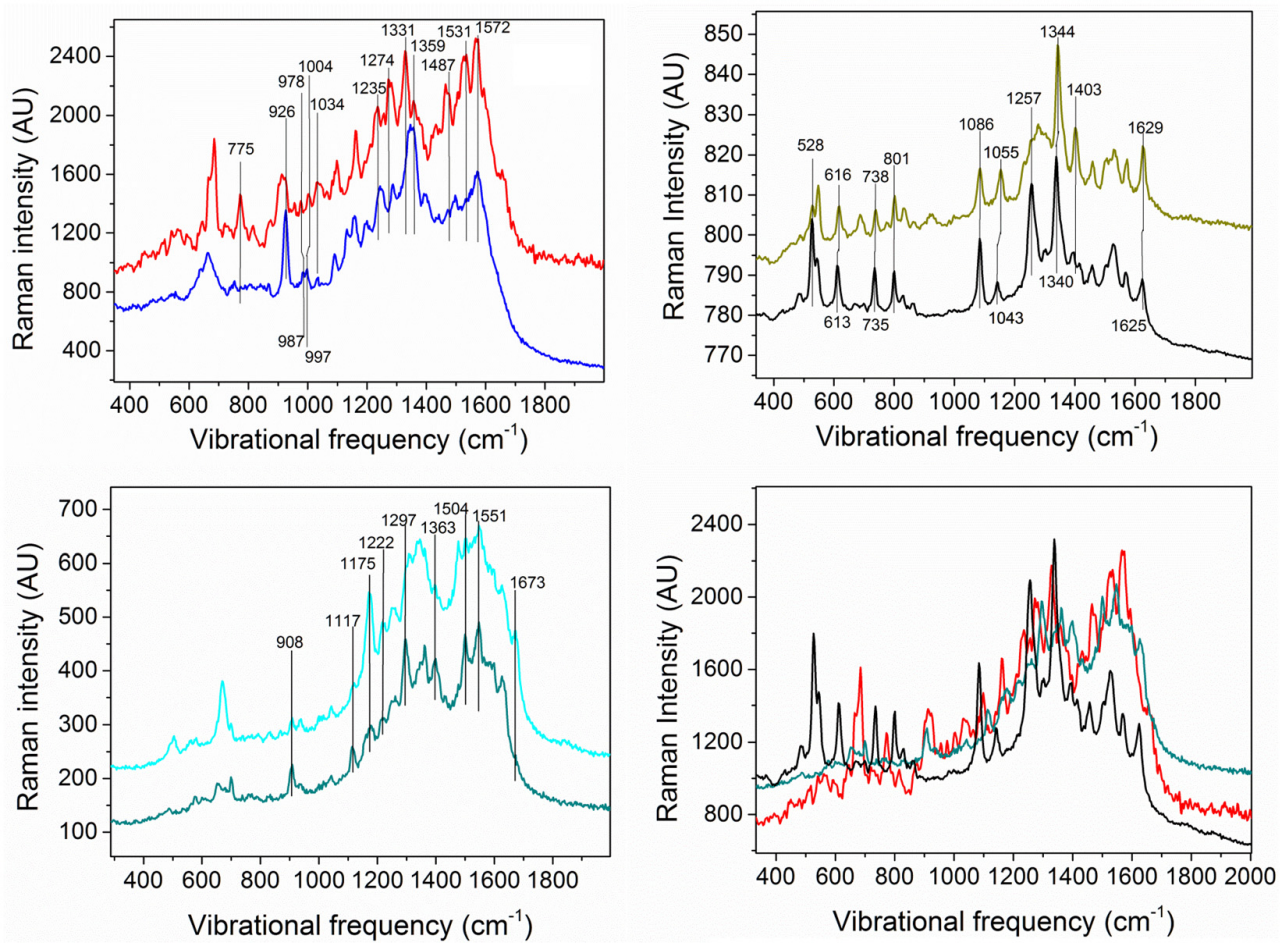
SERS spectra of expected biofilm component proxies. Each component was dissolved either in water (Sodium Alginate) or in phosphate buffer (hhcytc and riboflavin phosphate) and mixed with colloidal silver for Raman surface enhancement. a) reduced (red) and oxidized (blue) hhcytc; b) reduced (black) and oxidized (olive) riboflavin phosphate; c) two different SERS spectra of sodium alginate; d) spectra of reduced hhcytc, reduced riboflavin phosphate and sodium alginate overlaid, for comparison.

In order to identify the individual SERS spectra of pertinent components of the *S*. *oneidensis* biofilm, we first performed SECRaM analysis on several component proxies mixed with colloidal silver (Sigma Aldrich, Hamburg, Germany). The proxy component-colloidal silver mixture was analyzed under the same working conditions as the ones applied for biofilm analysis. The same excitation wavelength, laser intensity and setup were used, however without a Ag/AgCl patch. This was done, separately, for the following expected component proxies: horse heart cytochrome c (hhcytc) (Sigma Aldrich, Hamburg, Germany) in Phosphate buffer solution (PBS) (Roth, Karlsruhe, Germany); riboflavin phosphate (Sigma Aldrich, Hamburg, Germany) in PBS; hhcytc in PBS with excess sodium dithionite (Merck, Darmstadt, Germany); hhcytc in PBS with excess potassium hexacyanoferrate (III) (Sigma Aldrich, Hamburg, Germany); riboflavin phosphate in PBS with excess sodium dithionite; riboflavin phosphate in PBS with excess potassium hexacyanoferrate; an aqueous solution of sodium alginate (Sigma Aldrich, Hamburg, Germany); pure PBS; PBS with sodium dithionite; and PBS with potassium hexacyanoferrate. Sodium dithionite and potassium hexacyanoferrate, respectively, were used to obtain the reduced and oxidized hhcytc and riboflavin phosphate. Riboflavin phosphate, hhcytc and sodium alginate have been used as standard proxies for flavins, *cytochromes* and polysaccharides present in the biofilm, respectively. In the resulting proxy component SECRaM images, each pixel contained only some of all the Raman bands of the analyzed molecule, since in SERS the signal intensity is strongly dependent on the distance from the metallic surface and on molecular orientation, as only modes parallel to surface normal become enhanced [49,53,80]. This is true even for pure compounds if they are non-uniformly oriented, and especially for macromolecules such as hhcytc and alginate. To overcome this problem, we have averaged over all signal-containing pixels in each scan, to obtain the proxy component spectra shown in Fig 1. The peak at 1086 cm^-1^ in both the riboflavin phosphate and the hhcytc spectra is attributed to phosphate [81–84]. In Fig 1c two different spectra of sodium alginate appear, averaged on different signal-containing pixels of the same scan, because when averaging these two spectra, not all peaks are resolved. As seen in Fig 1d, all main expected components have coinciding peaks. Therefore a special approach had to be employed to analyze the SECRaM data in biofilm samples, as detailed below.

### SECRaM Data analysis

As seen from the individual component spectra (Fig 1), there are many coinciding or semi-coinciding peaks shared by *cytochromes*, flavins and polysaccharides. As explained above, in SERS peak intensity greatly depends on the distance from the surface and on the vibrational mode orientation [49,50], rendering data analysis algorithms that depend on peak intensity ratio, such as cluster analysis, not applicable to this type of heterogeneous sample, where the AgNp size and shape is non-uniform, and the components are anisotropically distributed in the sample. We therefore had to develop a different method for differentiating between the four following components analyzed in our sample: oxidized flavins, reduced flavins, polysaccharides and reduced+oxidized *cytochromes*. As seen in Fig 1a, reduced and oxidized *cytochromes* have very few peaks that can be resolved in order to differentiate between the two redox states, most notably the reduced *cytochrome* peak at 775 cm^-1^, absent from the oxidized spectrum. However, most other reduced and oxidized peaks coincide either with each other or with peaks of other components expected to be found in the biofilm. For example, the oxidized hhcytc peak at 1499 cm^-1^ coincides with a shoulder in the reduced hhcytc spectrum and with a peak in the alginate spectrum. The reduced hhcytc peak at 1432 cm^-1^ coincides with an alginate peak. The oxidized hhcytc peak at 1331 cm^-1^ coincides with both a shoulder in the reduced hhcytc spectrum and the alginate peak at 1337 cm^-1^ as well as its shoulder at 1328 cm^-1^. And while reduced hhcytc coincides with its oxidized counterpart only at 926 but not at 903 or 914 cm^-1^, the alginate peak at 909 cm^-1^ interferes with the latter two peaks. Therefore we have treated reduced and oxidized *cytochromes* together.

To differentiate between the four analyzed components (polysaccharides, *cytochromes*, reduced flavins and oxidized flavins), whose Raman peaks coincide in many cases (Fig 1d) the following approach was employed: Two types of Raman image were first generated from the biofilm SECRaM scan acquired on day 6. **Type I, unique peaks:** the sum of all sum filters for the peaks of each proxy component, which were removed from any other peaks or shoulders by at least 4 cm^-1^; **Type II, coinciding peaks of two components:** the sum of all sum filters for the coinciding peaks of each two proxy components i.e. the ones separated by less than 4 cm^-1^. These two types of Raman images were then individually binarized by taking the most intense 10% pixels of each image. This thresholding provided optimized chemical maps for all components, avoiding over-saturation and excessive coincidence of component-specific pixels. Average spectra were calculated from these binarized Raman images: one of each type for each analyzed biofilm component. For each of the four analyzed biofilm components (excluding phosphate, see below), the average spectra for unique peaks (Type I) were compared with those for coinciding peaks (Type II). This was performed as follows: (i) The sum of: Type I average spectrum for component A (e.g. *cytochromes*) + Type I average spectrum for component B (e.g. polysaccharides), was compared to the Type II average spectrum for the coinciding peaks of A and B. (ii) The sum of Type II average spectrum for the coinciding peaks of A and B + Type II average spectrum for the coinciding peaks of B and C (e.g. reduced flavins) was compared to the Type I average spectrum of component B. This was done for all component combinations. Any peak appearing in the two compared spectra in both (i) and (ii) was assigned to the respective analyzed biofilm component if it also appeared in the pure proxy component spectrum. In this way, seven to nine component-specific peaks were assigned to each analyzed biofilm component. These peaks are clearly marked in Fig 1. For a partial peak assignment list, see S1 Table. For phosphate, the typical peak at 1086 cm^-1^ appearing in the riboflavin phosphate and the hhcytc spectra was assigned [81–84].

Sum Raman images, consisting of the sum of all sum filters for the final analyzed component peaks, were produced for each scan. The top 10% most intense pixels in the resulting sum images were selected for the binarized chemical map images. 10% has been chosen, as it provided the clearest images. Lower thresholding has produced over-saturated chemical maps. The binarized images also served for creating an average spectrum for each individual component in the biofilm. SECRaM images summed over 1400–1600 cm^-1^ were obtained to show the Raman surface enhancement evolution in the biofilm.

### Sample fixation and drying for SEM-EDX

For SEM-EDX, samples on glass coverslip support (0.17 mm thick) were used. To both open the sample setup and reduce sample size to fit the SEM-EDX sample holder, a circle of ca. 7 mm diameter was drawn using a diamond scribe (TH.Geyer, Renningen, Germany) around the Ag/AgCl ink patch. Then the glass around the circle was removed and discarded, and the top coverslip was separated from the support coverslip and positioned face up. The biofilm on both the top and support coverslips was immediately fixed with 4% glutaraldehyde in cacoclydate buffer (Electron Microscopy Sciences, PA, USA) and was incubated overnight at 4°C. The next day, the fixing agent was rinsed off with Milli-Q water and subsequently stepwise solvent-exchanged with a graded series of acetone/water solutions (30%, 50%, 80%, 90%, 95%, 100%, each for 20 min). The sample was then critical point dried (Leica EM CPD300) and sputtered with a 15 nm Cr layer (Leica EM SCD500), following the procedure used by Ray et al [85]. Of the three replicates prepared for SEM-EDX analysis, 2 were fixed and analyzed seven days after sealing. One sample analyzed 14 days after sealing originates from an identical experiment prepared from the same strain a few months earlier.

### Electron-microscopy and Energy-Dispersive X-Ray Spectroscopy (SEM and EDX)

The morphology and structure of the samples were investigated by scanning electron-microscopy (SEM). A Zeiss Merlin VP field-emission SEM was used. In order to achieve high surface-sensitivity, the energy of the electron beam was set to 1.5 kV, unless otherwise specified, and the secondary electron (SE) detector was used. Working distance and beam current in imaging mode were approximately 1.5 mm and 14pA, respectively. The spatial resolution for imaging under these conditions was ca. 5 nm.

The SEM is equipped with a Bruker High-speed high-solid-angle XFlash^®^ FQ5060 QUAD SDD Detector (BRUKER Nano, Berlin, Germany), enabling a spatially-resolved elemental composition analysis of samples using energy-dispersive X-ray spectroscopy (EDX). In these experiments, X-rays are emitted during electronic transitions in atoms, in which tightly-bound electrons in core shells are expelled by the primary electron beam of the SEM. Owing to the X-ray energies characteristic to different atoms, EDX added elemental information to electron microscopy as follows: First, a full X-ray energy spectrum was recorded at each pixel of the image. Then the three-dimensional EDX data was reduced to a set of images which represented the spatial distribution of particular elements, at ca. 40 nm spatial resolution. This has been achieved by plotting the intensities of the X-ray energies of interest on two-dimensional false-color maps using the Bruker Esprit software library. In this study, electron beam acceleration of 5 kV was chosen for the EDX experiments in order to conveniently detect silver in the samples, using the L-alpha line at 2.98 keV. SEM measurements were also performed at 5 kV for comparison. The working distance and beam current in EDX mode were approximately 11 mm and 18 pA, respectively. Coinciding SEM and EDX images were superimposed using ImageJ.

To acquire SEM-EDX images of the bare Ag/AgCl patch, it was subjected to the same fixation, solvent exchange, critical point drying and sputtering procedures described above. To compare the SEM-EDX images of the biofilm to those of freshly deposited bacteria, a *S*. *oneidensis* culture in minimal medium (OD_600_ = 1.0) diluted to 50% in fresh medium was pipette deposited on the Ag/AgCl patch. However, the chamber was not sealed. Instead, after two minutes the patch with the freshly deposited bacteria was subjected to the same fixation, solvent exchange, critical point drying and sputtering procedures described above.

## Results and Discussion

### *Shewanella oneidensis* MR-1 produced AgNp-containing biofilm at an Ag/AgCl solid interface

**Fig 2:**
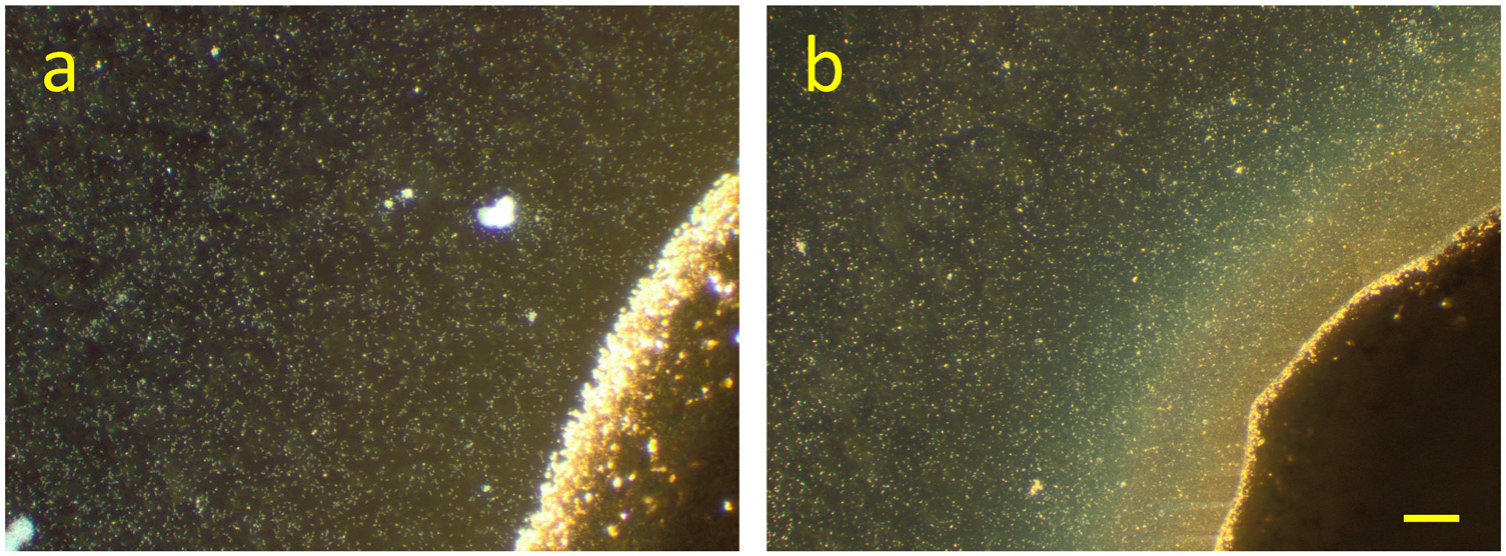
Dark field microscopy (x10) of *S*. *oneidensis* MR-1 at the Ag/AgCl solid interface. Bacteria appear as bright dots, and the Ag/AgCl patch is the dark area at bottom right corner. **a)** 30 min after sealing the setup, **b)** 7 days after sealing the setup: a brownish-orange light refracting substance has accumulated at the Ag/AgCl patch. Scale bar 50 μm.

The development of *S*. *oneidensis* MR-1 biofilms at a Ag/AgCl solid interface has been monitored in time by digital photography, light microscopy, SEM-EDX and SECRaM. The biofilms have already been visible to the naked eye after three days, and had a brownish hue with a silvery luster when tilted in the light (S1 Fig). In Fig 2, a part of the Ag/AgCl patch is shown, seven days after the setup was closed. In this dark field light microscopy image, the patch is seen as a dark area, while the microbial cells appear as bright dots. The biofilm can be observed as a brownish-orange light refracting substance at the patch. The color can be attributed to one or a combination of the following components, which absorb in the green: AgNp, *cytochromes*, and polysaccharides. To confirm that the feature seen in Fig 2b is indeed the result of bacterial colonization of the Ag/AgCl patch while using it as an electron acceptor, control experiments were performed (S2 Fig). In the abiotic control no such observations were made, and the Ag/AgCl patch remained unchanged. In the non-reducible ink control, the bacteria did not survive and only their debris could be seen, with no visible changes near the polymer patch.

In the soluble electron acceptor control, performed by adding disodium fumarate to the bacterial suspension, the bacteria survived and seem to have also precipitated AgNp and agglomerated around the Ag/AgCl patch; however, no light-refractive substance was observed (S2 Fig), indicating that a biofilm might not have been formed or was not as rich in polysaccharides. This observation may either indicate differences in biomass production rates due to a different energetic gain for bacterial growth with fumarate and Ag(I) compared to Ag(I) alone as the terminal electron acceptor; or that the bacteria might have chosen, while still respiring Ag(I) from the Ag/AgCl patch, not to build structures on it that would constrain fumarate diffusion to the cells. It has been previously shown that different multi-heme *cytochromes* in *Shewanella* are responsible for electron transfer to a variety of terminal electron acceptors (TEA), including fumarate, nitrate, Fe(III) and Mn(III/IV) oxides and their complexes, flavins and electrodes ([23,28] and references therein), however they all receive respiratory electrons from one protein, CymA, and many of them are expressed immediately upon the onset of anoxic conditions. This is apparently related to the *crp* gene expression [17]. Their function seems to be dependent on the small tetraheme *cytochrome* and/or fumarate reductase, either of which can mediate electron transfer to all of the abovementioned TEAs [28]. It is therefore clear that *Shewanella* can use different terminal electron acceptors in parallel, as observed here. Furthermore, it has been shown that diffusion within a *Shewanella* biofilm is restricted, especially near the surface [86].

**Fig 3:**
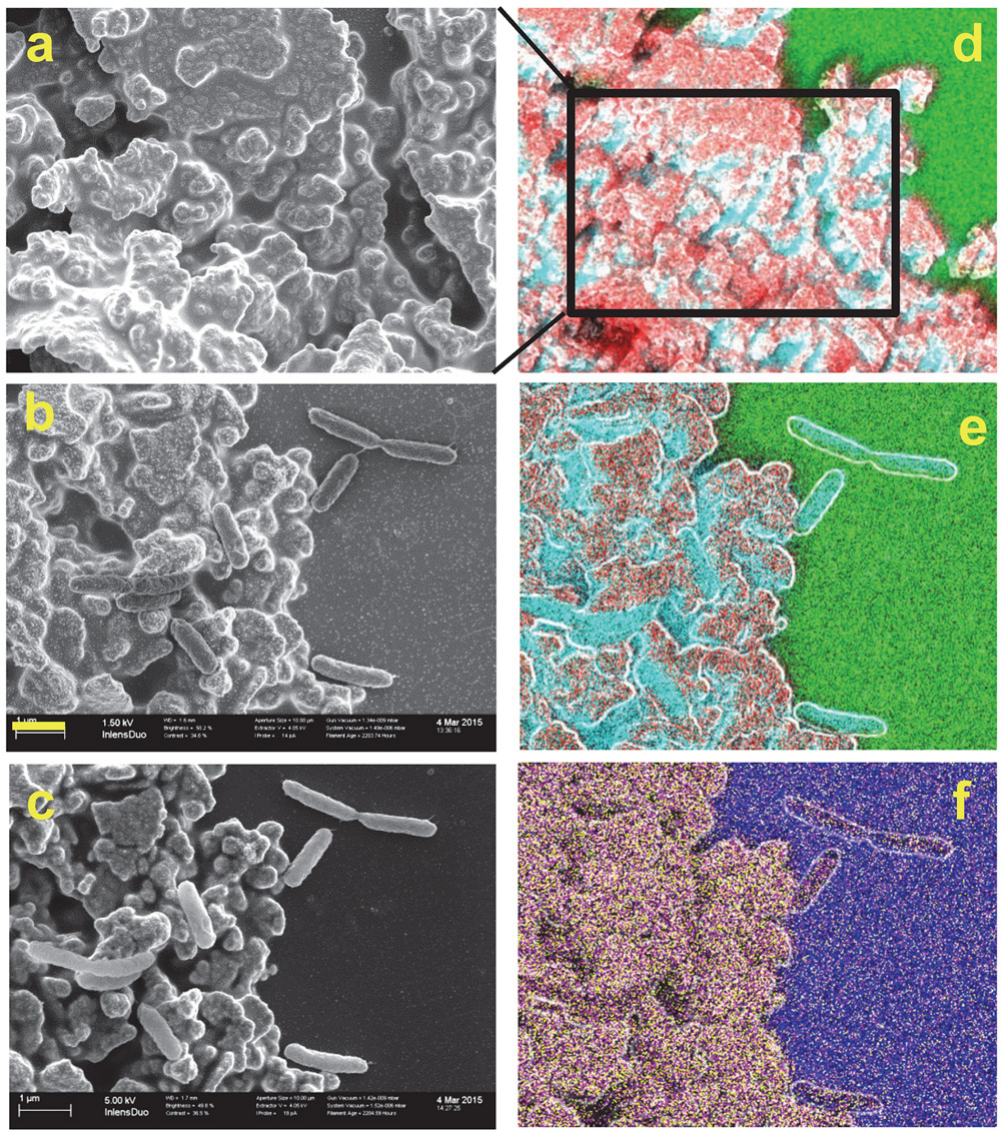
SEM-EDX analysis of a Ag/AgCl patch with (b, c, e, f) and without (a and d) freshly deposited *S*. *oneidensis* cells. Left column: SEM images, right column: overlay SEM-EDX images. Color code: Red: Ag, Cyan: C, Green: O, Magenta: P, Yellow: S, Blue: Si. Yellow bar is 1 μm and applies to all images but d. Images a and b were obtained at 1.5 kV and c to f at 5 kV, compare with Fig 4. kV stands for kilovolts and represents the acceleration voltage of the electron beam.

To obtain more information about the biofilm, SEM-EDX measurements have been performed on samples opened and chemically fixed 7 and 14 days after sealing the setup. For comparison, identical measurements have been performed on cured Ag/AgCl ink patches without bacteria and on Ag/AgCl patches with freshly deposited and immediately fixed *S*. *oneidensis* in minimal medium. In the SEM images we could observe the typical topographies of the bare Ag/AgCl patch and the expected morphology of planktonic cells (Fig 3b and 3c). After imaging by SEM, the elemental composition of the sample was recorded by SEM-EDX (Fig 3d, 3e and 3f). As seen in Fig 3, sulfur (S) and phosphorus (P) are abundant in the Ag/AgCl ink polymer matrix, while oxygen (O) and silicon (Si) are abundant in the glass support. Nitrogen could not be significantly detected in these EDX images, and therefore in the following figures carbon (C) is used as a marker for bacterial cells. For EDX peak assignment, see S3 Fig.

**Fig 4:**
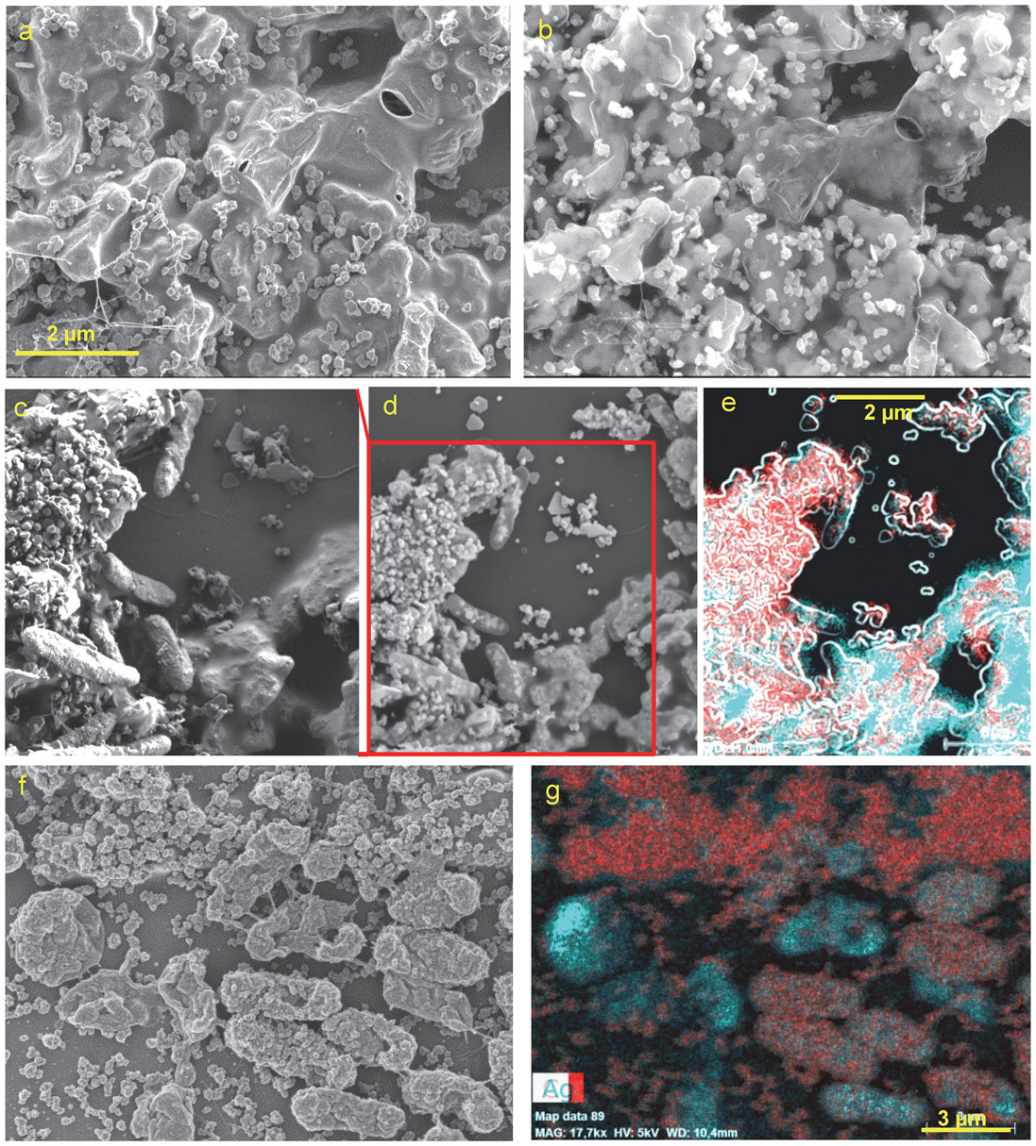
SEM-EDX images of 7 days (a-e) and 14 days (f-g) old biofilms. Scale bar 2 μm applicable for panels a, b and c (see bar at a) d, and e (see bar at e). For panels f and g scale bar is 3 μm (see bar at g). Panels a, c and f were acquired at 1.5 kV; d, e and g at 5 kV and b at 18 kV. In EDX images red is silver (Ag) and cyan is carbon (C). Image e is a superposition of the EDX color maps for Ag and C and a contour image produced from panel d using ImageJ plugin FeatureJ Edges. From panels a-e it is evident that AgNp in various shapes and sizes can be found both on the EPS and under the outer membrane of some bacteria (see text for more details). In panels f and g both the EPS and the outer membrane of some bacteria are completely covered with roughly spherical AgNp or agglomerations thereof. For a higher resolution segment of image f, see S4 Fig.

Using SEM-EDX analysis of the samples from days 7 and 14 we could observe the formation of an AgNp-containing biofilm at the Ag/AgCl patch (Fig 4). In Fig 4a and 4c, acquired with 1.5 kV electron beam acceleration, the EPS is observed covering the surface and obscuring the typical Ag/AgCl patch topography. This topography is revealed in the image acquired at 18 kV, where the electron beam can penetrate deeper, under the EPS contour (Fig 4b). In Fig 4a–4e, AgNp are seen in their previously documented shapes [87], as also previously reported for other AgNp-precipitating bacterial species, such as *Pseudomonas stutzeri* [88] and *Morganella psychrotolerans* [89]. In the samples reported here, the most abundant are circular AgNp of 110–190 nm diameter, triangular nanoprisms with a perpendicular bisector of 200–240 nm, and snipped nanoprisms (oblate unequal hexagons) of 250–400 nm. Prism thickness is ca. 20 nm, corresponding to a 1:10 to 1:20 aspect ratio. At this size-range, plasmon resonance for both triangular and snipped nanoprisms would be at >700 nm [90,91]. However, as edges become rounder, plasmon resonance becomes blue-shifted [87] until reaching a maximum near the excitation wavelength used in our study, 532 nm, as previously observed for semi-round particles of ca 125 nm [92], or as can be expected of larger but rounder particles, as the ones reported here. The way different AgNp shapes and sizes correspond to Raman surface enhancement at different exciting wavelength frequencies has been previously reported [93–95].

In Fig 4c, acquired at 1.5 kV, the outer membranes of the bacteria, as well as the EPS covering the Ag/AgCl patch (right bottom corner) are clearly seen, whereas in Fig 4d, the stronger 5 kV electron beam penetrates deeper, revealing the Ag/AgCl patch topography under the EPS, as well as AgNp found under the outer membrane of the bacteria, possibly in the periplasmic space. Triangular Ag nanoprisms are particularly easy to spot under the outer membrane of some bacteria. Similar findings have been reported for other species [88,96]. Some bacteria also touch AgNp externally. However, most of the AgNp precipitate seen in Fig 4a–4d is located on the EPS, suggesting that most of the SERS signal will likely originate from EPS components. This may also indicate that the bacterial Ag(I) reducing activity, i.e. electron transfer to extracellular IEA, is mediated by the EPS and its redox active components, namely *cytochromes* and flavins. It has been shown before that current production by *Shewanella* at an anode depends on EPS production [97].

After 14 days, both the EPS and many bacteria become completely covered with spherical AgNp of 60–100 nm diameter as well as rougher aggregates ranging between 140–220 nm, with surfaces featuring smaller spherical particles of 15–40 nm, spaced 4–6 nm (Fig 4f and 4g, and S4 Fig). This type of surface roughness has been shown to create considerable Raman enhancement at 514 nm [98].

### AgNp precipitated by *S*. *oneidensis* produce Raman enhancement for SECRaM

**Fig 5:**
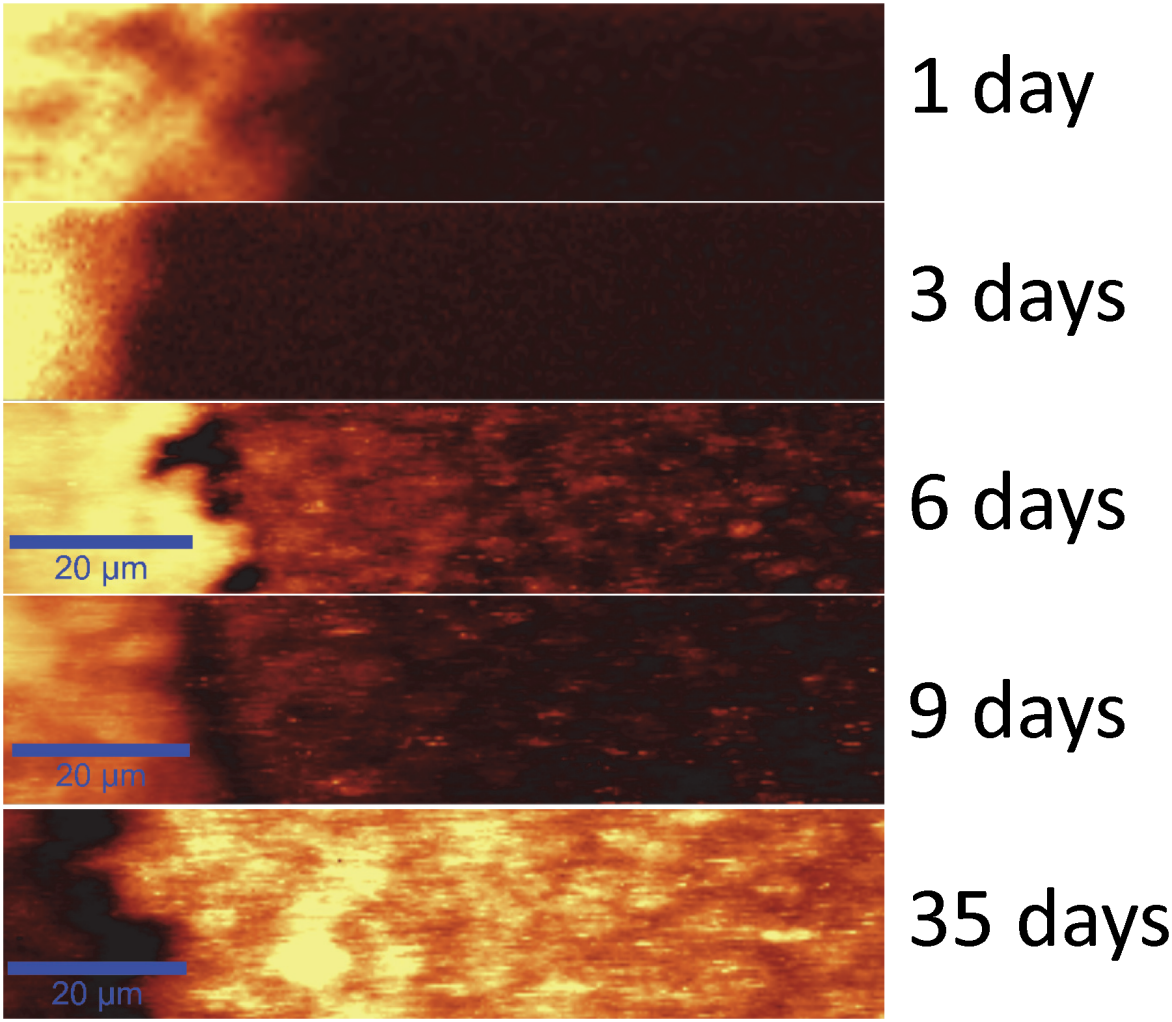
SECRaM time series of the *S*. *oneidensis* biofilm at the Ag/AgCl patch (patch seen on the left): 1, 3, 6, 9 and 35 days after sealing the setup. The Raman signal is summed over 1400–1600 cm^-1^. The 20 μm scale bar applies to all images. Intensity scale bar on left. All images normalized to 1000 a.u., without background subtraction. With time, Raman intensity hotspots develop where Raman-active biofilm components directly touch bio-produced AgNp. Raman hotspot intensity and distribution change with biofilm development, for discussion see text and Fig 6.

A time series of the development of Raman signal summed over 1400–1600 cm^-1^ in a typical sample is shown in Fig 5. The entire data series has been acquired under the same measurement conditions. On the left, the Ag/AgCl patch is visible as an area of high intensity due to Raman enhancement by the silver particles found in the Ag/AgCl patch itself. After 9 days the Raman intensity on the patch is decreased, and after five weeks a very poor signal is obtained in this region, possibly due to an excessive accumulation of light refracting molecules, such as flavins and EPS, hindering detection. On days 1 and 3 no Raman signal is detected outside the Ag/AgCl patch. However, on day 6 Raman signal hotspots appear where bio-deposited AgNp have reached the adequate size and shape for detecting the surface enhanced Raman signal when using a 532 nm excitation wavelength. The hotspot pattern changes as the biofilm develops, as can be seen on days 6, 9 and 35. Please note that an artifact is seen in the images, where the optical trapping effect exerted by the laser beam results in the dragging of solid or semi-solid objects, such as AgNp or perhaps even entire bacteria [68,99], in the wet biofilm for a few pixels, resulting in the appearance of horizontal lines that can clearly be seen in the SECRaM image.

**Fig 6:**
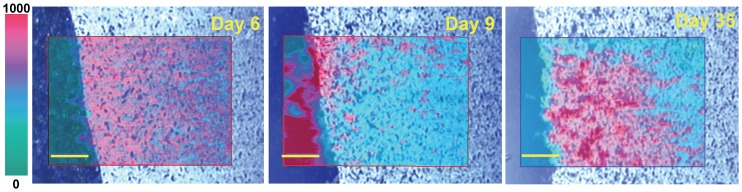
Time series of the *S*. *oneidensis* biofilm at the Ag/AgCl patch—surface enhanced Raman signal overlaid on light microscopy. 6, 9 and 35 days after sealing the setup. Ag/AgCl patch seen as dark area on the left. Grayscale: light microscopy, bright field, 20×. Black features are bacteria or bacterial aggregates, which have already precipitated Ag. Before precipitating Ag the bacteria are not visible in bright field, see also S1–S4 videos. Overlay: SECRaM image summed over 1400–1600 cm^-1^, intensity scale bar on left, all images normalized to 1000 a.u., without background subtraction. Yellow scale bar: 20 μm.

In Fig 6, larger SECRaM images are shown overlaid on bright field optical-microscopy images, where Ag-precipitating bacteria and bacterial aggregates are seen as dark spots (compare S1 and S2 Videos, where *S*. *oneidensis* bacteria are not seen in bright-field microscopy until they precipitate Ag). While on days 6 and 9 the Raman intensity hotspots do not coincide with the locations of such bacteria, on day 35 the most intense Raman signal is localized to bacterial aggregates. As seen above (Fig 4), this is explained by the observation that in samples fixed on day 7 for SEM-EDX, most AgNp were found on the EPS and not on bacterial outer membranes. In the sample fixed on day 14 the bacteria themselves show dense AgNp precipitates on their outer membranes. This also means that the biofilm chemical composition obtained by SECRaM will correspond on days 6 and 9 to the EPS and on day 35 to the vicinity of bacterial aggregates. However, since SERS signal is limited to ca. 2 nm from the AgNp surface [49,50], it is difficult to say whether the signal on day 35 stems from the bacterial outer membranes or from external components touching the precipitated AgNp.

All through the time series, more Raman intensity is detected near the Ag/AgCl patch, apparently because the probability of AgNp precipitation outside the patch depends on Ag(I) diffusion from AgCl particles in the patch to the rest of the biofilm. Since AgCl is very poorly soluble in water (Molar solubility 1.33×10^−5^ M [100]), it is also possible that flavins assist in its dissolution by chelation, as they do in the case of insoluble iron oxides [37,38]. Either way, since Ag(I) is being constantly consumed by bacterial respiration, the dissolution reaction for AgCl never reaches equilibrium, and Ag(I) is continuously released into the aqueous phase.

### Chemical mapping of MR-1 biofilm using SECRaM

**Fig 7:**
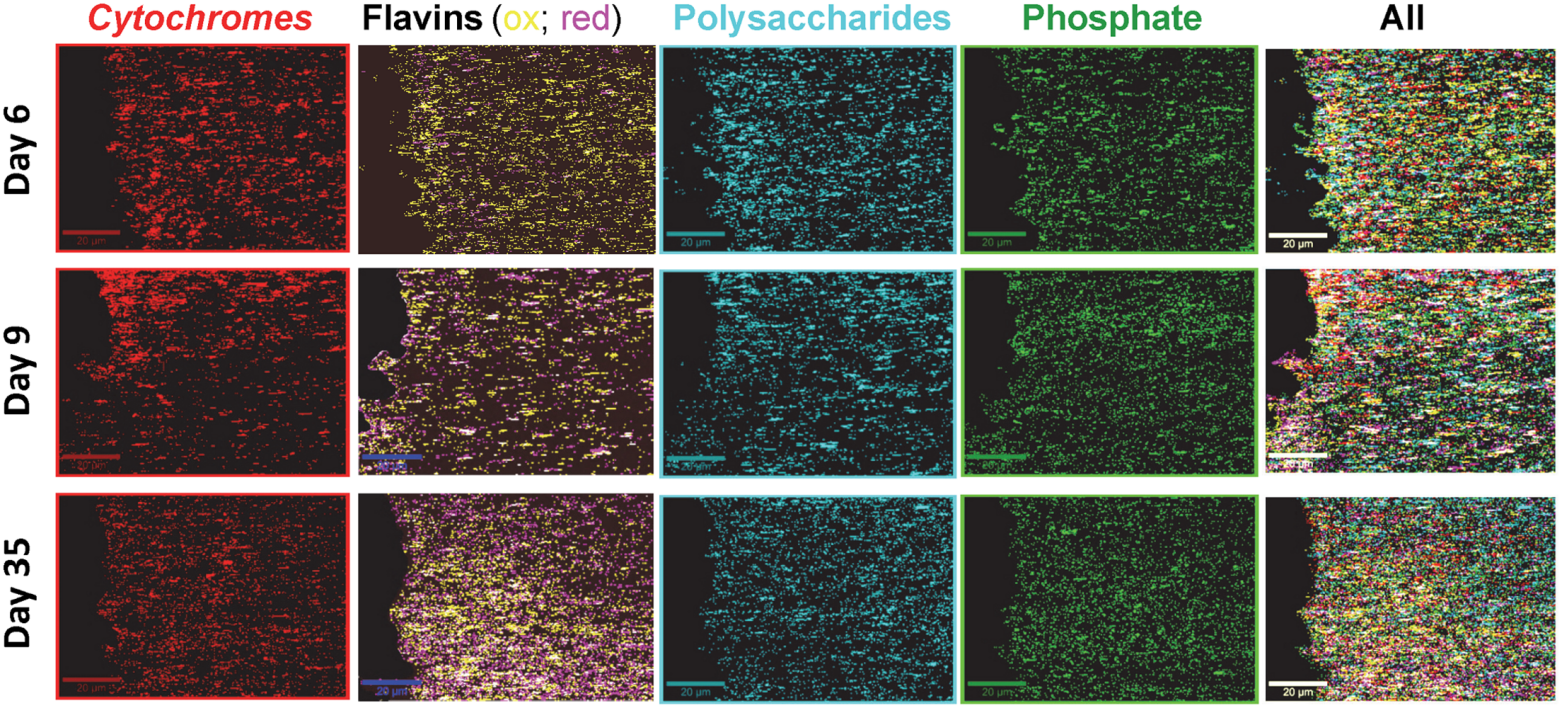
Chemical maps of a typical AgNp-precipitating *S*. *oneidensis* biofilm, produced by SECRaM. Top row: 6 days, middle row: 9 days, bottom row: 35 days after sealing the setup. Mapped biofilm components: Red: *cytochromes*, yellow: oxidized flavins, magenta: reduced flavins (white: reduced and oxidized flavins coinciding), cyan: polysaccharides, green: phosphate. Extreme right column is a superposition of all components. Scale bar: 20 μm.

After baseline subtraction of the SECRaM images and assignment of the different components (see Materials and Methods), intensity maps for each component have been obtained. For visualization and spectral averaging, the most intense 10% pixels in each intensity map have been chosen, and are shown in Fig 7 as single and superimposed component distribution maps. An *in-situ* time evolution of biofilm chemical composition has been obtained. Please note that pixels not belonging to the most intense 10% have been omitted, so that component coincidence is in reality more extensive than shown. Spectra averaged over the 10% most intense pixels for each component (i.e. the pixels seen in color in Fig 7) are shown in S5 Fig, overlaid with the spectrum of each pure proxy component (seen also in Fig 1). In the averaged biofilm spectra, peaks stemming from the pure components appear alongside peaks originating from other biofilm components coinciding within the same pixel.

**Fig 8:**
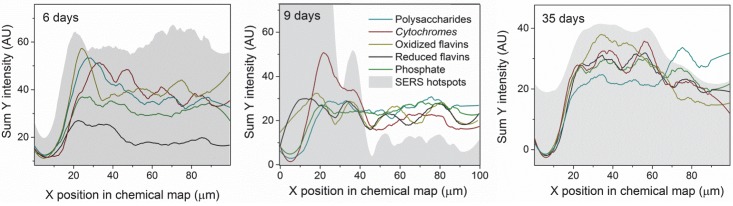
Component profiles, extracted using ImageJ from the chemical maps in Fig 7 and smoothed using Origin. Teal: polysaccharides; Maroon: *cytochromes*; Black: reduced flavins; Olive: oxidized flavins; Green: phosphates; Grey: SERS hotspots, normalized to fit the scale of the other components.

Distribution profiles for the different components in the sample are seen in Fig 8. On day 6, all components but phosphate are detected more intensely closer to the Ag/AgCl interface. On day 9, only *cytochromes* are detected more abundantly at the Ag/AgCl interface. On day 35, only flavins and *cytochromes* are detected more abundantly at the Ag/AgCl interface. *Cytochromes* and flavins follow the SERS hotspot distribution the most closely of all four components. For *cytochromes*, this is probably caused by a resonance of the Q-band region (green light absorption) with the excitation wavelength, which, multiplied with the surface enhancement for both the incident and the scattered light, results in a disproportionately higher enhancement of its signal [61,80]. Flavins, which absorb at ca. 370 nm and 450 nm [101], are not in resonance with the excitation wavelength used here, and the larger dependence of their detection on the SERS hotspot distribution, compared to phosphate and polysaccharides, may indicate that they tend to attach to AgNp in a favorable distance and/or orientation for detection using SERS. This observation is also supported by the exceptionally good S/N obtained for pure riboflavin phosphate mixed with colloidal silver (Fig 1). Flavins and *cytochromes* are therefore more abundantly detected near the Ag/AgCl solid interface, however this does not necessarily indicate that they are actually present there in higher concentrations. On day 35 flavins are detected more intensely than *cytochromes*, possibly indicating an accumulation of flavins in the biofilm. *Cytochromes*, being proteins, depend on a living cell to produce them and prevent them from denaturing, and therefore their concentration in the biofilm is limited by the number of living cells supported by the biofilm. Flavins, on the other hand, being much smaller and therefore more stable molecules, once discharged by the living bacterium can outlive it and be accumulated in the biofilm. Another possible explanation is that with time the flavins accumulate preferentially at the AgNp, thus preventing the enhancement of the *cytochrome* signal. Phosphate, a ubiquitous functionality of various biomolecules in both cells and the EPS [102], has been detected more or less uniformly across the biofilm at all stages (Fig 8). In contrast, polysaccharides have been first detected more abundantly next to the Ag/AgCl patch, then spread across the image to the right, and eventually their last distribution peak has moved out of the chemical map frame, possibly indicating a continued growth of the biofilm, with further accumulation of EPS in that direction. The oscillations visible in Fig 8, especially in the profile of the *cytochromes*, is an indication of the dynamical spatial organization of the biomass [103,104]. For day 6 the well resolved peaks point to a spatial clustering of the cells and EPS. As time advances, the biofilm population grows, first in proximity of the Ag/AgCl patch as can be seen from the enhanced *cytochrome* signal on day 9, and then the population becomes more homogeneous by day 35. Similar results have been observed in previously analyzed samples, produced in identical experiments prepared from the same strain at another date (an example is shown in S6 Fig).

As explained in the Materials and Methods section, it has not been possible to resolve reduced from oxidized *cytochromes* with the method used here. Thus, they were treated as one single component. However, since the *ν*_*15*_ pyrol breathing peak at 775 cm^-1^, typical of reduced *cytochromes* [72,105], is missing from the biofilm *cytochrome* averaged spectrum (S5 Fig, maroon), one may deduce that most of the Raman signal arising from *cytochromes* is accounted for by oxidized *cytochromes*. This is true for days 6, 9 and 35, and it may indicate that the *cytochromes* throughout the biofilm were maintained oxidized for the duration of the measurements by electron transfer to Ag(I). However, the dominance of the averaged spectra by oxidized, rather than reduced, *cytochromes* may also have two other explanations: (i) It may reflect the sensitivity of the UV/vis absorption spectrum of *cytochrome* to external conditions, such as pH, ionic strength and surface tension, especially in the vicinity of the excitation wavelength used here [106]. 532 nm is a wavelength on the *cytochrome* UV/Vis spectrum that is particularly sensitive to such conditions, meaning that under the as yet unknown microenvironmental conditions prevailing in the biofilm at different biofilm development stages and distances from the solid interface, oxidized *cytochromes* may be at a stronger resonance with the exciting wavelength than reduced *cytochromes*, resulting in a disproportionate enhancement of the oxidized *cytochrome* signal in our measurements, which may not indicate a higher concentration; (ii) Another possibility is that detection of *ν*_*15*_ pyrol breathing mode at 775 cm^-1^, belonging to the B_1g_ symmetry group, which is thus preferentially enhanced when the heme plane is oriented perpendicular to the AgNp surface [52,80], is suppressed under the experimental conditions described here, due to a possible preferential heme-parallel orientation of AgNp precipitated in the vicinity of *cytochromes*. Such preferential orientation may be deduced, for example, from recent X-ray diffraction data of undecaheme *cytochrome* crystalized in the presence of soluble Fe(III) chelates [23,26].

For flavins it is easier to discern reduced from oxidized, based on vibrational frequencies (Fig 1), enabling us to report the distribution of reduced vs. oxidized flavins in space and time (Fig 6). As seen, on day 6 more oxidized flavins were detected, but with time reduced flavins became more abundant, possibly indicating an increasing difficulty of the bacteria to find an adequate electron acceptor. This may arise from an increase in biofilm density next to the solid interface with time [86,107], brought about by the accumulation of cells, cell debris and EPS during the experiment. Such an accumulation of biofilm components at the Ag/AgCl patch may inhibit either or both flavin diffusion towards the poorly soluble Ag(I) salt, or Ag(I) diffusion away from the original patch to other parts of the biofilm. In both cases, this would result in less flavin (re)oxidation and an accumulation of reduced flavins.

## Conclusion and Outlook

We have shown that the development and chemical composition of a *S*. *oneidensis* MR-1 biofilm formed at a Ag/AgCl solid interface can be followed *in situ* using surface enhanced confocal Raman microscopy, without the need to open the system and add soluble Ag(I) salts and/or abrasive reduction agents, as had been done before to investigate bacteria using SERS. This has provided the first chance, to our knowledge, to perform a spatially resolved *in situ* SERS time series of an undisturbed electroactive biofilm, repeatedly revisiting the same location of the intact biofilm at a solid interface under permanent anaerobiosis. Furthermore, the use of a Raman enhancing support, such as roughened electrode or patterned glass slides, has been avoided, allowing the SERS analysis of positions other than the bottom of the biofilm. This is also the first time, to our knowledge, that a concomitant chemical analysis of four pertinent *Shewanella* biofilm components with both spatial and temporal resolution is performed. In the future, such measurements can be performed in 3D and with a higher temporal resolution, supplying a wealth of information about these greatly important but still to be understood biofilms. Moreover, the application of SECRaM using bio-precipitated noble metal particles can be extended from *Shewanella* biofilms to AgNp and AuNp precipitation in bioremediation [108] and nanoparticle biosynthesis applications, including the removal of Ag(I) from mining environments [109] and photographic waste [110], precipitation of AuNp from Au(III) contaminated water by *Pseudomonas aeruginosa* biofilms [111], removal of various metal species from waste electronic scrap leachate by *Desulfovibrio desulfuricans* [112], and production of AgNp by *Aspergillus flavus* [113], *Morganella sp*. [89,114], *Lactobacillus* [96], various cyanobacteria [115] and *Pseudomonas stutzeri* [88]. The method presented here will open new ways of analysis by chemical mapping over time in 3D and *in situ*, without disturbing the involved microorganisms, for a wide range of applications.

## Supporting Information

S1 Fig Digital images of S. oneidensis MR-1 biofilm at the cured Ag/AgCl ink patch. Images recorded using an LG G3 mobile phone back digital camera. Left: the experimental setup consisting of a cured Ag/AgCl ink patch on a standard microscope slide, where *S*. *oneidensis* bacteria in defined medium were deposited and then covered with a standard cover slip. Silicone vacuum grease is used as both spacer and sealant to keep the setup air-tight. Middle and right columns: biofilm development as seen by the naked eye. On day 1 the Ag/AgCl patch is light beige in color. With time, it becomes darker, and a brownish biofilm visibly grows around it. When tilted in the light, the brownish biofilm exhibits a silvery luster, indicating the precipitation of Ag particles inside it. Sample was photographed 1, 3, 6, and 35 days after sealing the setup.
(PDF)

S2 Fig Dark field microscopy images (x10) of control experiments. Top: Abiotic control, performed in the absence of bacteria (setup contains Ag/AgCl patch and minimal medium, but no bacteria). No changes to the Ag/AgCl patch observed (compare Fig 1). Middle: non-reducible ink control with dielectric polymer instead of Ag/AgCl ink (setup contains cured dielectric polymer patch and *S*. *oneidensis* in minimal medium, without Ag/AgCl or any supplementary electron acceptors). The bacteria did not survive and did not settle at the dielectric polymer. Bottom: Soluble electron acceptor control, where 20 mM fumarate were added to the original setup. The bacteria survived and aggregated around the Ag/AgCl patch, but no brownish light-refracting material was deposited (compare Fig 1).
(PDF)

S3 Fig EDX peak assignment, performed by the Bruker Esprit software supplied with the Bruker Quantax detector, based on its spectral library. Left: averaged over a 2x2 (μm)2 area of original Ag/AgCl patch covered with EPS such as seen on the right bottom of Fig 3c–3e. Right: averaged over a 0.8x0.8 (μm)2 area of a Shewanella bacterium lying directly on the glass support. Both acquired on a sample fixed 7 days after sealing the setup.
(PDF)

S4 Fig Higher resolution image of a detail from Fig 3f. (PDF)

S5 Fig Raman spectra for pure proxy components vs. biofilm spectral averages. Red, olive, gray, blue&teal: SERS spectra of pure proxy components with colloidal Ag for hhcytc (reduced+oxidized), oxidized and reduced riboflavin phosphate and sodium alginate, respectively, as seen in Fig 6. Maroon, yellow, black, cyan: the corresponding average spectra for each component in the biofilm, from day 6, averaged over the 10% most intense pixels where the respective individual component has been detected. The 10% most intense pixels are seen in the chemical maps at the top row of Fig 7. In the averaged biofilm spectra, peaks of the corresponding individual component can be seen alongside other peaks from other components enhanced within the same pixel.
(PDF)

S6 Fig Figures analogous to Figs 6, 7 and 8, for a replicate experiment. Bacteria of the same strain, prepared and analyzed at another point in time, in the same way described in the article. The results are very similar to the ones discussed in the text.
(PDF)

S1 Table Raman peak assignment. Unassigned peaks were detected in pure component analysis (see Fig 1) but lack assignment in literature. If reduced (red) and oxidized (ox) species can be differentiated, it is mentioned in the table. For molecular schemes, please see references.
(PDF)

S1 Video Bright field (20×) videos of the system, 1 day after sealing. The bacteria are not visible in bright field microscopy.
(AVI)

S2 Video Bright field (20×) videos of the system, 6 days after sealing. When the bacteria started to precipitate AgNp they became visible in bright field microscopy. Twitching activity is seen.
(AVI)

S3 Video Bright field (20×) videos of the system, 9 days after sealing. When the bacteria started to precipitate AgNp they became visible in bright field microscopy. Twitching activity is seen.
(AVI)

S4 Video Bright field (20×) videos of the system, 35 days after sealing. With time, the bacteria organize in aggregates.
(AVI)
