# Correction: *In Situ* Analysis of a Silver Nanoparticle-Precipitating *Shewanella* Biofilm by Surface Enhanced Confocal Raman Microscopy

**DOI:** 10.1371/journal.pone.0199344

**Published:** 2018-06-14

**Authors:** Gal Schkolnik, Matthias Schmidt, Marco G. Mazza, Falk Harnisch, Niculina Musat

The following information is missing from the Funding section: MGM also gratefully acknowledges support from the Deutsche Forschungsgemeinschaft (SFB 937, project A20).
