# Supplementary material for: In Situ Analysis of a Silver Nanoparticle-Precipitating Shewanella Biofilm by Surface Enhanced Confocal Raman Microscopy
Source: PLoS One. 2015 Dec 28;10(12):e0145871. doi: 10.1371/journal.pone.0145871 (PMC4692441; doi:10.1371/journal.pone.0145871)
Supplement: S1 Fig — Images recorded using an LG G3 mobile phone back digital camera. Left: the experimental setup consisting of a cured Ag/AgCl ink patch on a standard microscope slide, where S. oneidensis bacteria in defined medium were deposited and then covered with a standard cover slip. Silicone vacuum grease is used as both spacer and sealant to keep the setup air-tight. Middle and right columns: biofilm development as seen by the naked eye. On day 1 the Ag/AgCl patch is light beige in color. With time, it becomes darker, and a brownish biofilm visibly grows around it. When tilted in the light, the brownish biofilm exhibits a silvery luster, indicating the precipitation of Ag particles inside it. Sample was photographed 1, 3, 6, and 35 days after sealing the setup. (PDF) [file pone.0145871.s001.pdf]

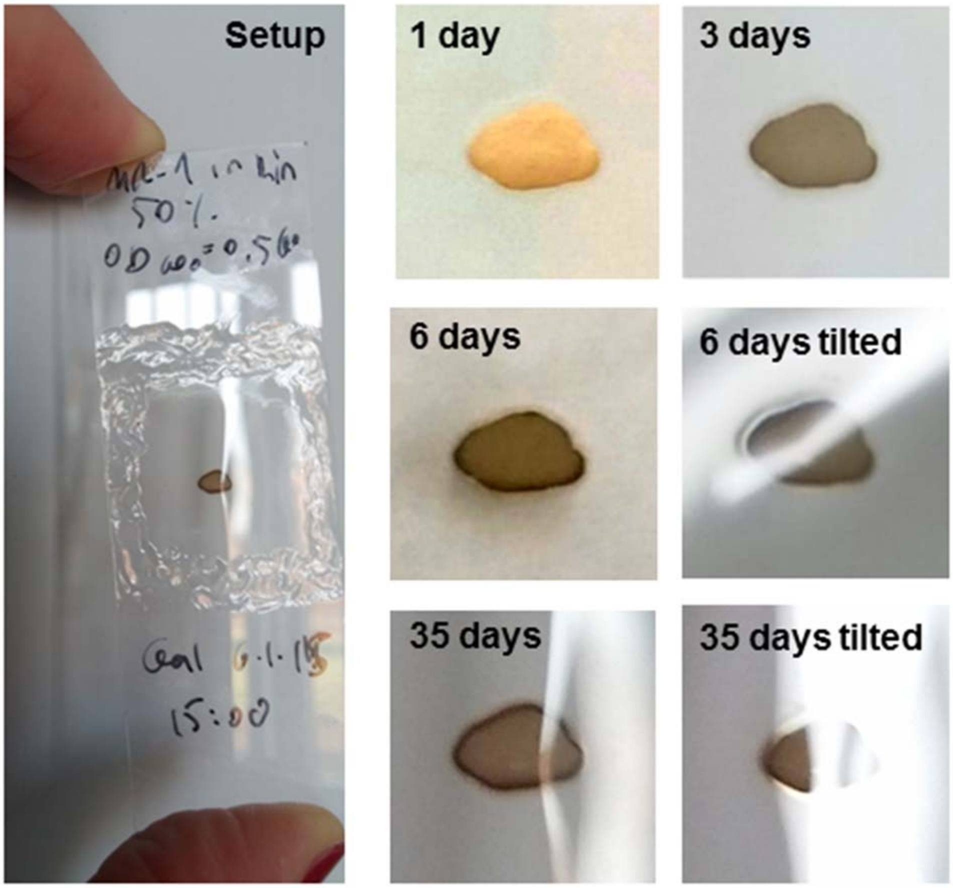

S1 Figure. Digital images of S. oneidensis MR-1 biofilm at the cured Ag/AgCl ink patch. Images recorded using an LG G3 mobile phone back digital camera. Left: the experimental setup consisting of a cured Ag/AgCl ink patch on a standard microscope slide, where *S. oneidensis* bacteria in defined medium were deposited and then covered with a standard cover slip. Silicone vacuum grease is used as both spacer and sealant to keep the setup air-tight. Middle and right columns: biofilm development as seen by the naked eye. On day 1 the Ag/AgCl patch is light beige in color. With time, it becomes darker, and a brownish biofilm visibly grows around it. When tilted in the light, the brownish biofilm exhibits a silvery luster, indicating the precipitation of Ag particles inside it. Sample was photographed 1, 3, 6, and 35 days after sealing the setup.
