# Supplementary material for: In Situ Analysis of a Silver Nanoparticle-Precipitating Shewanella Biofilm by Surface Enhanced Confocal Raman Microscopy
Source: PLoS One. 2015 Dec 28;10(12):e0145871. doi: 10.1371/journal.pone.0145871 (PMC4692441; doi:10.1371/journal.pone.0145871)
Supplement: S2 Fig — Top: Abiotic control, performed in the absence of bacteria (setup contains Ag/AgCl patch and minimal medium, but no bacteria). No changes to the Ag/AgCl patch observed (compare Fig 1). Middle: non-reducible ink control with dielectric polymer instead of Ag/AgCl ink (setup contains cured dielectric polymer patch and S. oneidensis in minimal medium, without Ag/AgCl or any supplementary electron acceptors). The bacteria did not survive and did not settle at the dielectric polymer. Bottom: Soluble electron acceptor control, where 20 mM fumarate were added to the original setup. The bacteria survived and aggregated around the Ag/AgCl patch, but no brownish light-refracting material was deposited (compare Fig 1). (PDF) [file pone.0145871.s002.pdf]

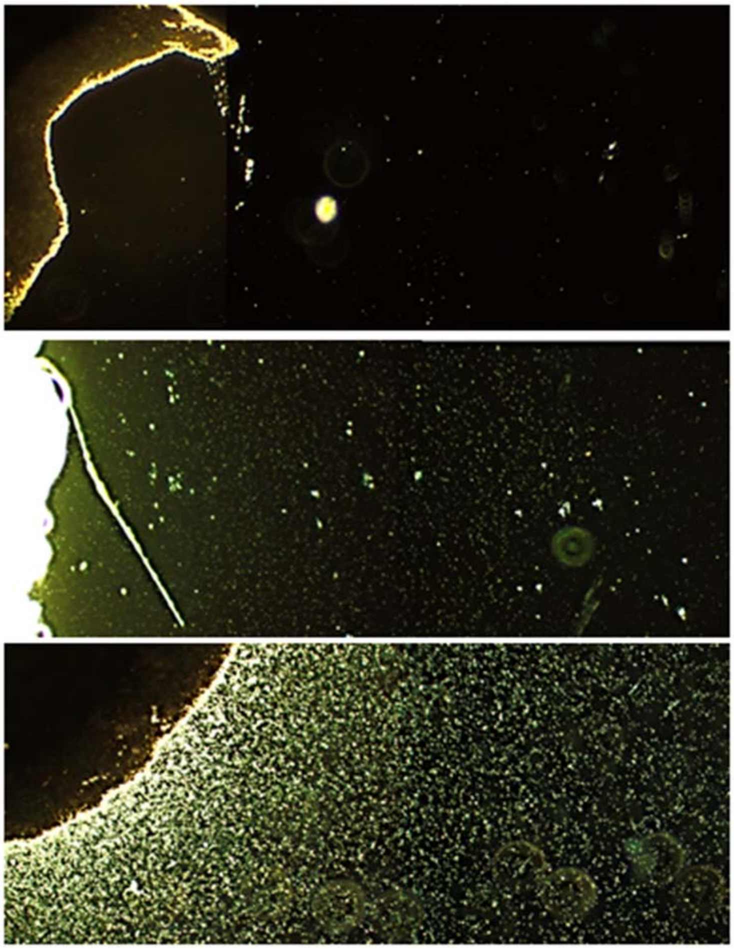

S2 Figure. Dark field microscopy images (x10) of control experiments. **Top:** Abiotic control, performed in the absence of bacteria (setup contains Ag/AgCl patch and minimal medium, but no bacteria). No changes to the Ag/AgCl patch observed (compare Figure 1). **Middle:** non-reducible ink control with dielectric polymer instead of Ag/AgCl ink (setup contains cured dielectric polymer patch and *S. oneidensis* in minimal medium, without Ag/AgCl or any supplementary electron acceptors). The bacteria did not survive and did not settle at the dielectric polymer. **Bottom:** Soluble electron acceptor control, where 20 mM fumarate were added to the original setup. The bacteria survived and aggregated around the Ag/AgCl patch, but no brownish light-refracting material was deposited (compare Fig. 1).
