# Supplementary material for: In Situ Analysis of a Silver Nanoparticle-Precipitating Shewanella Biofilm by Surface Enhanced Confocal Raman Microscopy
Source: PLoS One. 2015 Dec 28;10(12):e0145871. doi: 10.1371/journal.pone.0145871 (PMC4692441; doi:10.1371/journal.pone.0145871)
Supplement: S3 Fig — Left: averaged over a 2x2 (μm)2 area of original Ag/AgCl patch covered with EPS such as seen on the right bottom of Fig 3c–3e. Right: averaged over a 0.8x0.8 (μm)2 area of a Shewanella bacterium lying directly on the glass support. Both acquired on a sample fixed 7 days after sealing the setup. (PDF) [file pone.0145871.s003.pdf]

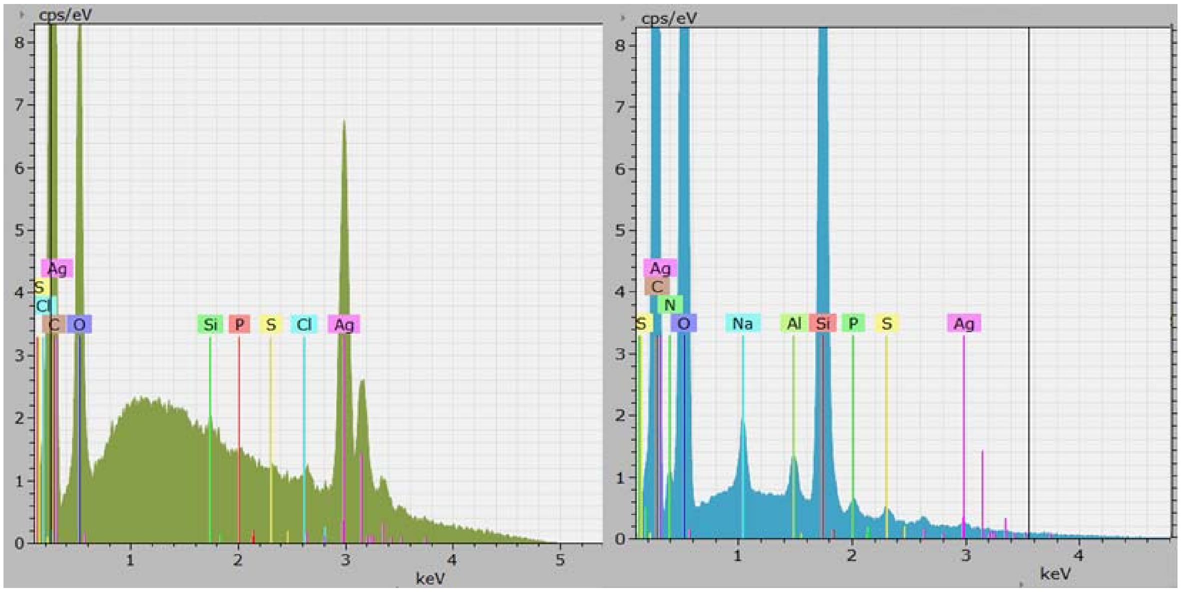

S3 Figure. EDX peak assignment, performed by the Bruker Esprit software supplied with the Bruker Quantax detector, based on its spectral library. **Left**: averaged over a 2x2 (µm)^2 area of original Ag/AgCl patch covered with EPS such as seen on the right bottom of Fig. 4c-e. **Right:** averaged over a 0.8x0.8 (µm)^2 area of a *Shewanella* bacterium lying directly on the glass support. Both acquired on a sample fixed 7 days after sealing the setup.
