# Supplementary figures and images for: In Situ Analysis of a Silver Nanoparticle-Precipitating Shewanella Biofilm by Surface Enhanced Confocal Raman Microscopy
Source: PLoS One. 2015 Dec 28;10(12):e0145871. doi: 10.1371/journal.pone.0145871 (PMC4692441; doi:10.1371/journal.pone.0145871)

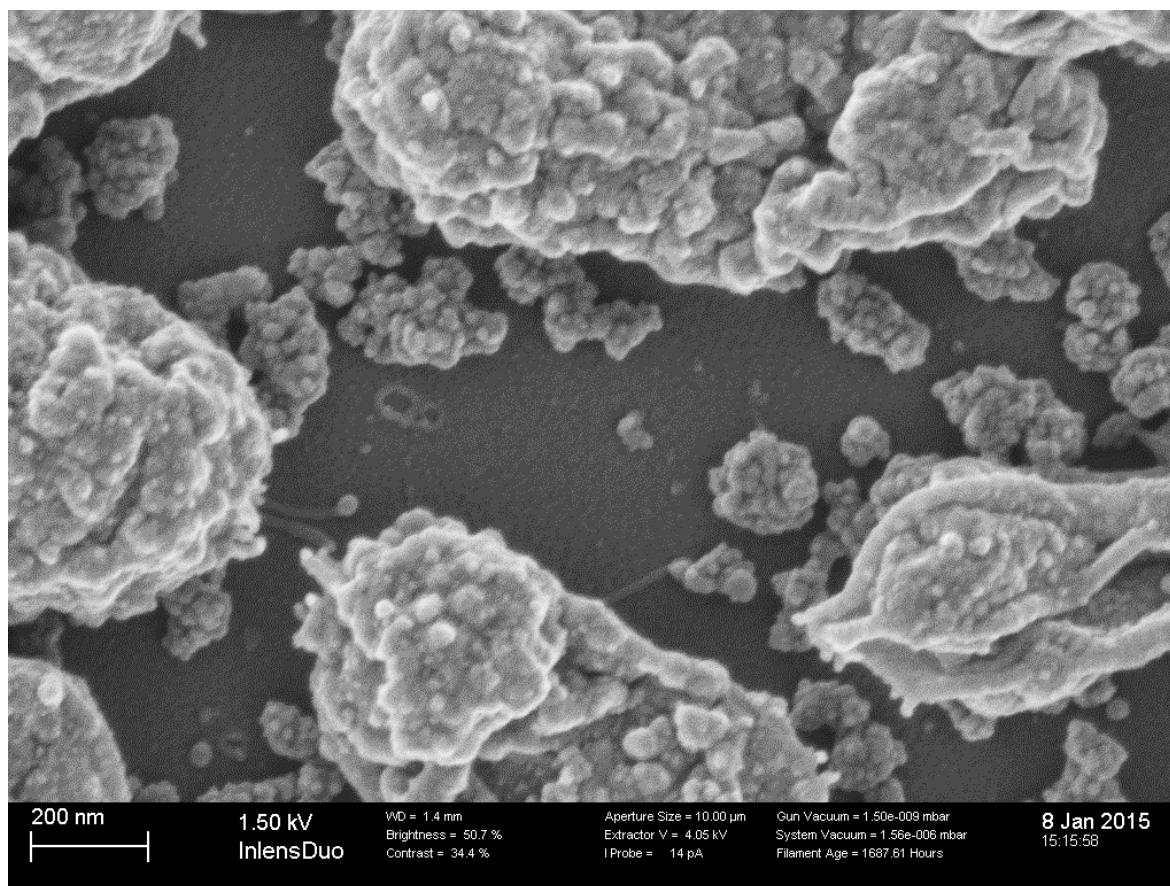

**S4 Figure.** Higher resolution image of a detail from Figure 3f.

Supplement: S4 Fig — (PDF) [file pone.0145871.s004.pdf]
