# Supplementary material for: In Situ Analysis of a Silver Nanoparticle-Precipitating Shewanella Biofilm by Surface Enhanced Confocal Raman Microscopy
Source: PLoS One. 2015 Dec 28;10(12):e0145871. doi: 10.1371/journal.pone.0145871 (PMC4692441; doi:10.1371/journal.pone.0145871)
Supplement: S5 Fig — Red, olive, gray, blue&teal: SERS spectra of pure proxy components with colloidal Ag for hhcytc (reduced+oxidized), oxidized and reduced riboflavin phosphate and sodium alginate, respectively, as seen in Fig 6. Maroon, yellow, black, cyan: the corresponding average spectra for each component in the biofilm, from day 6, averaged over the 10% most intense pixels where the respective individual component has been detected. The 10% most intense pixels are seen in the chemical maps at the top row of Fig 7. In the averaged biofilm spectra, peaks of the corresponding individual component can be seen alongside other peaks from other components enhanced within the same pixel. (PDF) [file pone.0145871.s005.pdf]

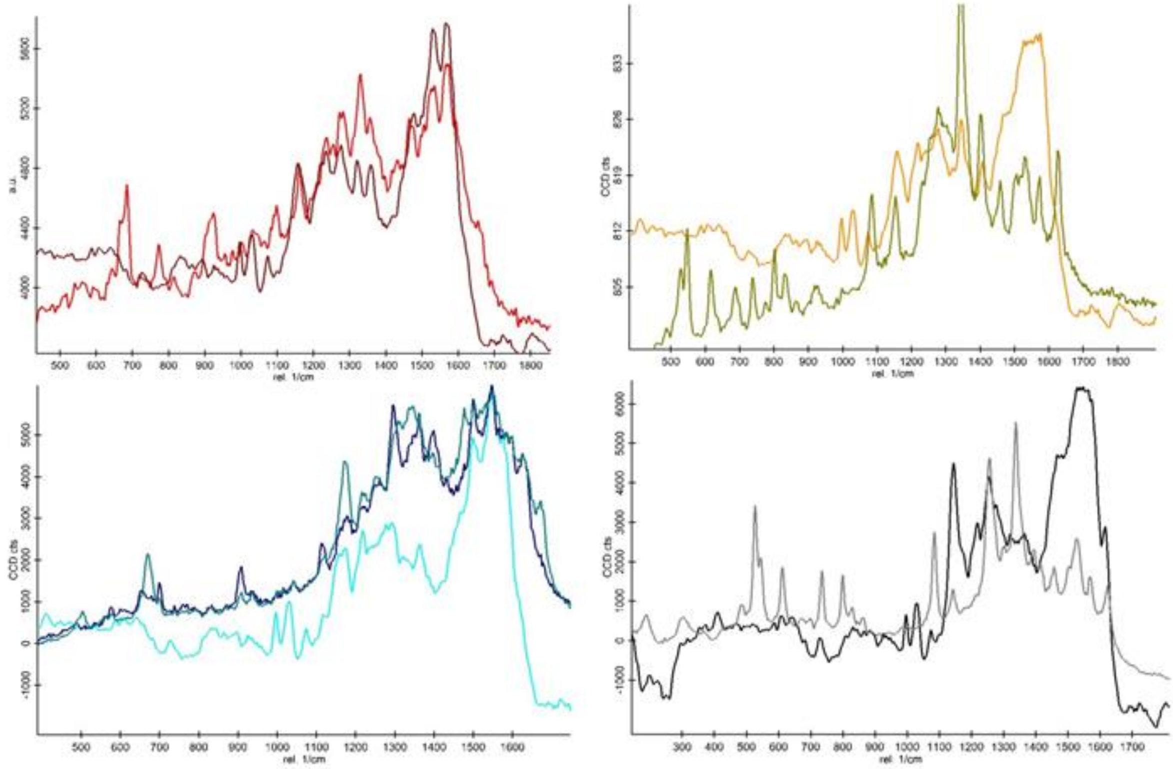

S5 Figure. Raman spectra for pure proxy components vs. biofilm spectral averages. Red, olive, gray, blue&teal: SERS spectra of pure proxy components with colloidal Ag for hhcytc (reduced+oxidized), oxidized and reduced riboflavin phosphate and sodium alginate, respectively, as seen in Fig. 6. Maroon, yellow, black, cyan: the corresponding average spectra for each component in the biofilm, from day 6, averaged over the 10% most intense pixels where the respective individual component has been detected. The 10% most intense pixels are seen in the chemical maps at the top row of Fig. 7. In the averaged biofilm spectra, peaks of the corresponding individual component can be seen alongside other peaks from other components enhanced within the same pixel.
