# Supplementary material for: In Situ Analysis of a Silver Nanoparticle-Precipitating Shewanella Biofilm by Surface Enhanced Confocal Raman Microscopy
Source: PLoS One. 2015 Dec 28;10(12):e0145871. doi: 10.1371/journal.pone.0145871 (PMC4692441; doi:10.1371/journal.pone.0145871)
Supplement: S6 Fig — Bacteria of the same strain, prepared and analyzed at another point in time, in the same way described in the article. The results are very similar to the ones discussed in the text. (PDF) [file pone.0145871.s006.pdf]

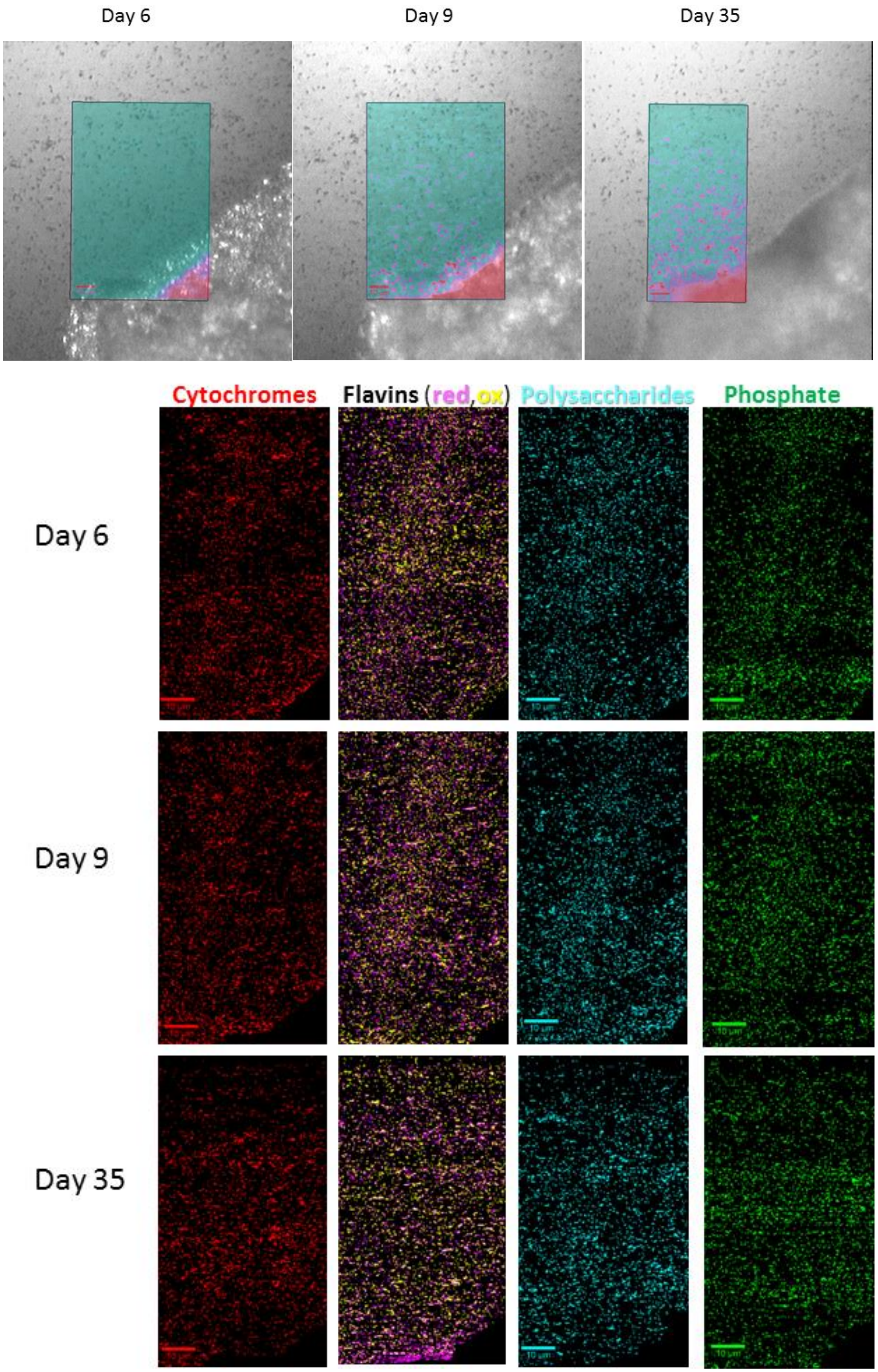

Day 6
Day 9
Day 35
Cytochromes
Flavins (red, ox)
Polysaccharides
Phosphate
Day 6
Day 9
Day 35

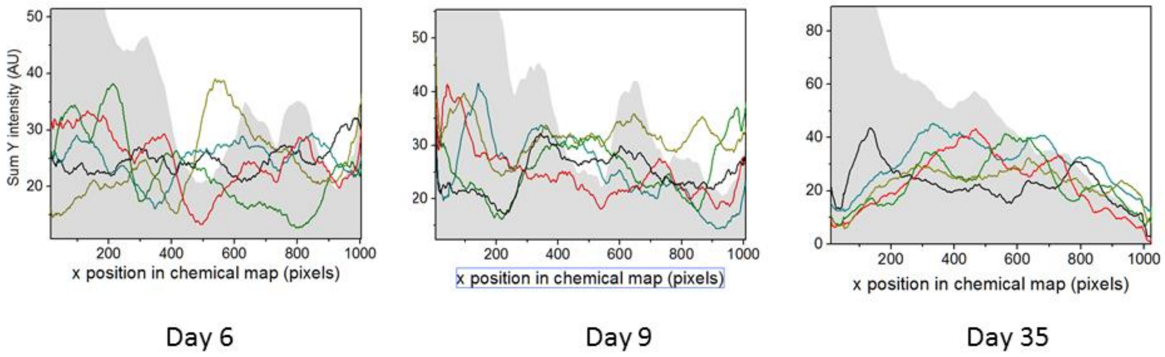

S6 Figure. Figures analogous to Figs 6, 7 and 8, for a replicate experiment. Bacteria of the same strain, prepared and analyzed at another point in time, in the same way described in the article. The results are very similar to the ones discussed in the text.
