# Supplementary material for: In Situ Analysis of a Silver Nanoparticle-Precipitating Shewanella Biofilm by Surface Enhanced Confocal Raman Microscopy
Source: PLoS One. 2015 Dec 28;10(12):e0145871. doi: 10.1371/journal.pone.0145871 (PMC4692441; doi:10.1371/journal.pone.0145871)
Supplement: S1 Table — Unassigned peaks were detected in pure component analysis (see Fig 1) but lack assignment in literature. If reduced (red) and oxidized (ox) species can be differentiated, it is mentioned in the table. For molecular schemes, please see references. (PDF) [file pone.0145871.s007.pdf]

S1 Table. Raman peak assignment. Unassigned peaks were detected in pure component analysis (see Fig. 1) but lack assignment in literature. If reduced (red) and oxidized (ox) species can be differentiated, it is mentioned in the table. For molecular schemes, please see references.

Component	Raman shift (cm^{-1})	Assignment	Reference
Cytochromes	775 (red)	ν_{15} pyrol breathing	(1,2)
	978 (red) 987 (ox)	$\nu(C_c,C_d)$	(2)
	1034	ν_{31}	(2)
	1235	ν_{13}	(1,2)
	1274 (red)		
	1331	ν_{21} or $\delta(C_aH)$	(1,2)
	1359	ν_4	(1,2)
	1478		
	1531		
	1572	ν_{19}	(2)
Flavins (red/ox)	528/-		
	613/616		
	736/738	Ring I deform	(3)
	801/801	Ring I deform, C_6-H bend, N_3-H bend	(3)
	1143/1153	Ring I and Ring III modes	(3)
	1257/-	Ring I modes, N_5-H, C_6-H, C_9-H bend<br>If resonance: C_{5a}-N_5, N_3-C_4<br>No resonance: C_2-N_3 str	(3,4)
	1340/1344	Adenine bend, framework vibrations	(3,4)
	-/1403	N_3-C_4 str, C_{10a}-C_{4a} stretch	(4)
	1625/1629	C_6-C_7 str, C_8-C_9 str, N_5-C_{4a} str	(4)
Alginate	908	ν_{sym} C-O-C	(5)
	1117		
	1175	ν_{asym} C-O-C	(5)
	1222	$\nu_{C\text{-}O}$	(5)
	1297	$\delta_{C\text{-}H}$	(5)
	1363	$\delta_{C\text{-}H}$	(5)
	1504	ν_{asym} COO^-	(5)
	1551	ν_{asym} COO^-	(5)
	1673		
Phosphate	1086	ν_{sym} PO_2^-	(6,7)

S1 Table references

1. Virdis B, Millo D, Donose BC, Batstone DJ. Real-time measurements of the redox states of c-type cytochromes in electroactive biofilms: A confocal resonance raman microscopy study. PLoS One. 2014;9(2).

2. Hu S, Morris I, Singh J. Complete assignment of cytochrome c resonance Raman spectra via enzymic reconstitution with isotopically labeled hemes. J Am … [Internet]. 1993;(c):12446–58. Available from: http://pubs.acs.org/doi/abs/10.1021/ja00079a028

3. Zheng Y, Carey PR, Palfey B a. Raman spectrum of fully reduced flavin. J Raman Spectrosc [Internet]. 2004 Jul 28 [cited 2015 Aug 31];35(7):521–4. Available from: http://doi.wiley.com/10.1002/jrs.1218

4. Weigel a., Dobryakov a., Klaumünzer B, Sajadi M, Saalfrank P, Ernsting NP. Femtosecond stimulated Raman spectroscopy of flavin after optical excitation. J Phys Chem B. 2011;115(13):3656–80.

5. Campos-Vallette MM, Chandía NP, Clavijo E, Leal D, Matsuhiro B, Osorio-Román IO, et al. Characterization of sodium alginate and its block fractions by surface-enhanced Raman spectroscopy. J Raman Spectrosc [Internet]. 2010 [cited 2015 Aug 31];41(7):758–63. Available from: http://doi.wiley.com/10.1002/jrs.2517

6. Xu L-J, Lei Z-C, Li J, Zong C, Yang CJ, Ren B. Label-Free Surface-Enhanced Raman Spectroscopy Detection of DNA with Single-Base Sensitivity. J Am Chem Soc [Internet]. 2015;150414092441008. Available from: http://pubs.acs.org/doi/abs/10.1021/jacs.5b01426

7. Greaves SJ, Griffith WP. Surface-Enhanced Raman Scattering (SERS) from Silver Colloids of Vanadate, Phosphate and Arsenate. 1988;19(December 1987):503–7.
